# D-Glucuronolactone Supplementation Enhances Production Performance, Eggshell Quality, and Liver Health in Laying Hens

**DOI:** 10.3390/ani15091317

**Published:** 2025-05-01

**Authors:** Yiru Shen, Zhiqiang Miao, Yuqi Zheng, Yuanyang Dong, Miaomiao Han, Chenxuan Huang, Rui Bai, Chengqiang Xia, Shourong Shi, Jianhui Li

**Affiliations:** 1 Laboratory of Poultry Production, College of Animal Science, Shanxi Agricultural University, Jinzhong 030801, China; shenyiru929@126.com (Y.S.); mzhq1981@163.com (Z.M.); 18335442965@163.com (Y.Z.); yuanyangdongemail@126.com (Y.D.); h_miaomiao2019@163.com (M.H.); huangchenxuanvip@163.com (C.H.); chinabairui@sxau.edu.cn (R.B.); xiachengqiang126@163.com (C.X.); 2 Jiangsu Institute of Poultry Sciences, Yangzhou 225125, China

**Keywords:** glucuronolactone, Hy-Line brown hen, laying rate, liver fat, eggshell color

## Abstract

The liver of hens bears a very heavy workload in handling lipogenesis as well as carbohydrate, protein, and other nutrient metabolism to maintain a high laying rate. D-glucuronolactone (DGL) is a naturally occurring chemical metabolite produced from glucose and exhibits a hepatoprotective effect as a food ingredient. This study aimed to evaluate the effect of DGL on production performance, egg quality, and liver health in laying hens during their peak period. Four hundred and eighty Hy-Line brown hens (aged 42 weeks), with comparable high-laying performance, were randomly assigned to four groups, with each group consisting of eight replicates. The control group was fed a basal diet, while the treatment groups were supplemented with DGL at 70, 140, and 280 mg/kg in the basal diet, respectively. The results indicated that DGL supplementation significantly increases the laying rate and egg mass of hens (*p* < 0.05). The brown color of the eggshell was significantly deepened by DGL administration (*p* < 0.05). Additionally, DGL supplementation significantly decreased the serum levels of aspartate aminotransferase and γ-glutamyl transpeptidase (*p* < 0.05). The liver index, liver fat, and triglyceride levels in the liver had also significantly decreased (*p* < 0.05). Liver histopathological results showed that the control group exhibited yellow coloration with large gaps and numerous vacuolar cells, while DGL supplementation improved the compactness of liver parenchyma. Antioxidant activity was enhanced, showing a significant increase in the activity of total antioxidant capacity and catalase in the liver (*p* < 0.05). The inflammatory response was alleviated, with a significant decrease in expressions of interleukin-β, tumor necrosis factor-α, interferon-α, interleukin-6, and interleukin-8 genes in the liver (*p* < 0.05). Moreover, DGL supplementation significantly reduced cell apoptosis-related genes, including factor-related apoptosis, Caspase 7, and BCL2 associated X protein in the liver (*p* < 0.05). Overall, these findings indicate that dietary DGL enhanced the laying performance of hens by maintaining liver health, with the best results achieved at a dose of 280 mg/kg in this trial.

## Simple Summary

Health status of the liver is pivotal in hens for maintaining their high performance. D-glucuronolactone (DGL) is widely used as a hepatoprotective supplement in humans, which may have a positive effect on liver health and laying performance of hens. Our findings reveal that DGL supplementation at a dose of 280 mg/kg increases laying performance of hens and enhances the liver health status, evidenced by significantly improved parameters including liver functions, histopathological results, antioxidant capacity, inflammatory response, and cell apoptosis.

## 1. Introduction

Hy-Line brown layers are among the most commonly raised breeds of laying hens. They are well known for their prolonged and stable egg-laying performance. These hens maintain a high laying rate for almost 40 weeks during the laying period. However, their production declines sharply around 60 weeks as they enter the late-laying phase. The liver contributes to over 90% of de novo lipogenesis for prolonged periods to sustain yolk synthesis during this high-production phase. To meet the nutritional demands of daily egg production, the liver of laying hens bears a much heavier workload than that of mammals, handling lipogenesis as well as carbohydrate, protein, and other nutrient metabolism [1]. Commercial hens in intensive egg production systems have high egg-laying genetic potential. Their higher metabolic rates, compared to hens in extensive breeding, demand more precise nutritional care [2]. As hens age, lipid peroxidation and protein oxidation occur [3,4], antioxidant recovery diminishes [5,6], hepatocyte growth is inhibited, and liver tissue repair is delayed [7], leading to reduced production performance [6,8]. Excess lipids, metabolic toxins, and harmful reactive oxygen species (ROS) accumulate in the liver during peak production and continue through the aging process. These challenges raise concerns about their impact on liver function in peak-laying hens, potentially causing irreversible liver damage and declining production rates in aged hens. Meanwhile, in the traditional perception of animal nutrition, corn and soybean meals have long been regarded as the proper used raw materials in livestock and poultry diets. However, the pronounced vulnerability of corn to mold contamination and the persistent presence of anti-nutritional factors in soybean meals present a significant concern in harboring the potential risk of increasing the liver’s workload [9].

D-glucuronolactone (DGL), a naturally occurring metabolite derived from glucose, is present in all connective tissues in humans. Historically, DGL has been used as a hepatoprotective agent to alleviate liver metabolism burdens, supporting the detoxification of chemicals such as drugs and metabolic waste [10]. Furthermore, DGL has been reported to inhibit tumor promoters associated with carcinogens and mitigate their harmful effects [11]. Studies have shown that DGL possesses notable inhibitory effects on α-amylase and glucosidase activity, aiding glycogen depletion [12,13].

In this research, DGL was reported to possess favorable inhibitory activities of α-amylase and glucosidase and help with glycogen depletion, which could significantly improve the uptake of glucose and the synthesis of glycogen [14]. Prior research suggests that the long-term consumption of DGL at safe doses is tolerable and may support organ health, particularly liver health. This suggests potential benefits for peak-laying hens enduring heavy metabolic burdens. However, few studies have investigated the effects of DGL on poultry either in vivo or in vitro. Yu et al. [15] demonstrated that DGL could mitigate the hepatotoxic effects of ochratoxin A in chicken hepatocytes, while another study focused on broilers, which had a different research purpose [16]. Apart from these findings, there is limited data on the use of DGL in poultry, particularly egg-laying hens.

As the liver status of hens after prolonged egg production warrants further attention, we hypothesized that the hepatoprotective effects of DGL could promote liver health in egg-laying hens, potentially enhancing their laying performance. This study evaluated the effects of DGL on liver health in hens raised under normal rearing conditions. Laying performance and egg quality were assessed to determine the comprehensive benefits of DGL as a potential feed additive for the poultry industry.

## 2. Materials and Methods

### 2.1. Experiment Design and Animal Husbandry

Four hundred and eighty Hy-Line brown hens at 42 weeks with similar body weight, and individual laying rate higher than 90% in the last 2 months, were randomly divided into 4 groups with 8 replicates (15 hens per replicate). The hens were housed in step cages systems (23 × 40 × 40 cm each cage). In this trial, all hens were raised in a ventilated laying house with a stable temperature at 20 ± 3 °C and stable humidity level at 50 ± 5%. The photoperiod in this house is 16 h of daily light with 30 Lux of light intensity. The hens were provided ad libitum access to water and feed. Eggs were collected at 2 pm every day.

After a 1 week adaptation, hens were given a basal (CON) or DGL-added (70,140,280 mg/kg diet, G1-3, respectively) diet for 12 weeks. DGL (purity ≥ 95%) was supplied by Howtian pharmaceutical Co. Ltd. in Shandong province of China. The adding dosage of DGL was conducted according to the recommendation level published in European Food Safety Authority (EFSA) in adult humans [17]. The adding dosage of the DGL in this trial may be calculated as follows: (14 mg/kg BW/day) × 2 kg BW/(100 g feed intake) = 280 mg/kg diet, which was designed as the highest level for hens in this trial. Table 1 presents the composition and nutritional values of the basal diet. DGL was presented as a solid powder form and was premixed step-by-step in corn flours before being added in the basal diet.

### 2.2. Laying Performance

Laying performance parameters include egg laying rate (%), average egg weight (AEW, g), average daily feed intake (ADFI, g/hen per d), egg mass (g/hen per d), and feed conversion ratio (FCR), which were recorded and calculated weekly. The FCR was calculated as feed consumption/total egg weight. Meanwhile, the number of chickens death were also recorded every day to calculate the mortality rate.

### 2.3. Egg Quality

At the end of the experiment, a total of 160 normal eggs were randomly selected from each dietary treatment group for the assessment of egg quality traits. Specifically, this selection comprised 40 eggs per group, with 5 eggs being taken from each replicate. Egg-shape index was measured with a digital caliper and calculated using the following formula: egg transverse span/egg longitudinal span. Eggshell strength was determined using the Egg Force Reader, while the yolk color and Haugh unit were determined using an egg analyzer. The two machines were from ORKA Food Technology, Ltd., Ramat Hasharon, Israel. Shell thickness was determined using a gauge from Guanglu Measuring Instrument Co., Ltd. (Guilin, China) at air cell, equator, and sharp end of the eggs. The average of the shell thickness was calculated. Shell color was determined using a colorimeter (Konica Minolta, Tokyo, Japan).

### 2.4. Sample Collection

At the end of the experiment, one hen was randomly selected and weighed from each replicate. 1.5 mL blood samples from the wing were collected and centrifuged at 3500 rpm for 10 min to collect the serum, which was stored at −20 °C for later detection. Birds were sacrificed by jugular bleeding, while the whole liver was removed, photographed, and weighed. Part of the liver tissues were fixed in 4% neutral-buffered paraformaldehyde for hematoxylin–eosin (HE) staining. After that, the others were rapidly frozen in liquid nitrogen and then stored at −80 °C.

### 2.5. Clinical Blood Parameters

Clinical biochemistry measurements were obtained from the serum. The levels of total bilirubin (TBIL), alanine aminotransferase (ALT), aspartate aminotransferase (AST), alkaline phosphatase (ALP), and glutamyl transferase (GGT) were measured using an automatic biochemical analyzer (CS380, Dirui, Changchun, China).

### 2.6. Liver Index

The liver index (%) was calculated as [liver weight (g)/live weight of hen (g)] × 100.

### 2.7. Hepatic Lipid Accumulation

The Soxhlet extraction method was used to determine the fat content in the liver [18]. The level of triglyceride (TG) and total cholesterol (TC) were determined using a kit from Nanjing Jian-cheng Bioengineering Institute (Nanjing, China).

### 2.8. Histological Examination of the Liver Tissue

Liver samples were dehydrated and embedded in paraffin before sectioning and HE staining. The stained tissues were carefully observed using a microscope (DM4000b, Leica, Deerfield, IL, USA) (LAS X software, Version 3.5.5, Leica). To illustrate the impacts of DGL interventions, histopathological alterations were classified into three distinct levels of liver damage according to the previous study [19]. The higher the score value means the healthier the liver.

### 2.9. Antioxidant Capacity

Liver samples were homogenized in phosphate-buffered solution (PBS) and centrifuged to obtain liver homogenate. The protein concentration in homogenate was determined using a kit from Nanjing Jian-cheng Bioengineering Institute (Nanjing, China). After pretreatment, superoxide dismutase (SOD), catalase (CAT), glutathione peroxidase (GSH-Px), and total antioxidant capacity (T-AOC) activities were measured using the commercial kit (Nanjing Jiancheng Institute of Bioengineering, Nanjing, Jiangsu, China).

### 2.10. Hepatic Gene Expression Analysis

The TRIzol (TianGen, DP421, Beijing, China) method was used to extract RNA from liver tissues, as described in our previous study [20]. Primer information is listed in Table 2. The mRNA levels were normalized based on the levels of β-actin. 2^−∆∆Ct^ approach was used to calculate the relative expression levels of inflammatory response and cell apoptosis genes.

### 2.11. Statistical Analysis

Data collected in this trial were analyzed using SPSS 26.0 (SPSS Inc., Chicago, IL, USA). A one-way analysis of variance (ANOVA) was conducted and followed by Duncan’s multiple comparison test, linear, and quadratic regression analysis. Data were presented as the means with pooled standard error of mean (SEM). Differences were declared to be statistically significant at *p* < 0.05.

## 3. Results

### 3.1. Effect of DGL on Laying Performance

The results of the laying performance are shown in Table 3. At 8~12 weeks of the trial, the laying rate of hens were significantly increased by DGL administration (*p* < 0.05), which fit a linear model (*p* = 0.021, R^2^ = 0.286). The laying rate of the hens in the whole trial were significantly increased by DGL administration (*p* < 0.05), which fit a linear model (*p* = 0.002, R^2^ = 0.377).

At 5~8 weeks of the trial, the AEW of the laying hens had significantly increased due to DGL administration (*p* < 0.05), which fit a quadratic model (*p* = 0.029, R^2^ = 0.198). At 9~12 weeks of the trial, the AEW of the laying hens had significantly increased due to DGL administration (*p* < 0.05). The AEW of the hens in the whole trial had significantly increased due to DGL administration (*p* < 0.05), which fit a quadratic model (*p* = 0.015, R^2^ = 0.179).

At 1~4 weeks and 5~8 weeks of the trial, the egg mass of laying hens had significantly increased due to DGL administration (*p* < 0.05). The egg mass of the hens in the whole trial had significantly increased due to DGL administration (*p* < 0.05), which fit a linear model (*p* = 0.039, R^2^ = 0.134).

Dietary DGL administration showed no significant effect on the ADFI or FCR of hens throughout the whole trial period (*p* > 0.05). No chicken deaths were observed throughout the whole trial period.

### 3.2. Effect of DGL on Egg Quality

The results of the egg quality are shown in Table 4. The brown color of eggshell in the last week was significantly deepen due to dietary DGL administration (*p* < 0.05), which fit a linear model (*p* < 0.001, R^2^ = 0.261). DGL administration showed no significant effect on the other parameters of egg quality (*p* > 0.05).

### 3.3. Effect of DGL on Blood Biochemical Parameters

Results of the blood biochemical parameters are shown in Table 5. The AST level in blood had significantly decreased due to DGL administration (*p* < 0.05), which fit a linear model (*p* = 0.001, R^2^ = 0.305). The GGT level in blood had significantly decreased due to DGL administration (*p* < 0.05), which fit a linear model (*p* < 0.001, R^2^ = 0.131). DGL administration showed no significant effect on the other parameters of blood biochemical (*p* > 0.05).

### 3.4. Effect of DGL on Liver Indexes and Liver Lipids

Results of liver indexes and liver lipids are shown in Table 6. The liver index had significantly decreased due to DGL administration (*p* < 0.05), which fit a quadratic model (*p* = 0.007, R^2^ = 0.267). The liver fat had significantly decreased due to DGL administration (*p* < 0.05), which fit a liner model (*p* = 0.001, R^2^ = 0.176). The TG level in the liver had significantly decreased due to DGL administration (*p* < 0.05). DGL administration showed no significant effect on the TC level of the liver (*p* > 0.05).

### 3.5. Effect of DGL on Liver Histopathological Changes

**Figure 1:**
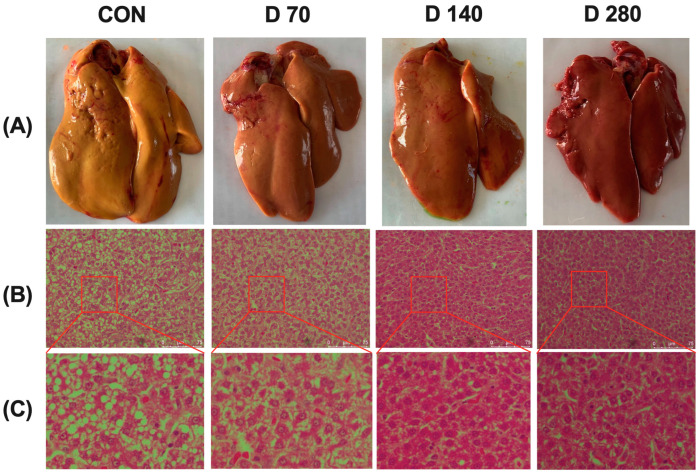
Images showing the changes in the liver at the end of the experimental period. (**A**) Representative images of the liver tissue from the four groups. (**B**) Liver histology images post hematoxylin and eosin (H and E) staining from the four groups. (**C**) The inset from (**B**) is shown in an enlarged view. Abbreviations: CON, basal diet group; D 70, D 140, and D 280, basal diet supplemented with 70, 140, and 280 mg/kg of D-glucuronolactone, respectively. The same as in the figures below.

Results of the liver histopathological changes are shown in Figure 1. The representative images of liver tissues are shown in Figure 1A. The liver of hens in CON group appeared in khaki color, while that of the hens in the DGL group appeared normal and kermesinus in color, especially in group D280. Representative images of HE-stained liver tissues are shown in Figure 1B,C. The liver parenchyma in the DGL treatment group was compact, while that in the control group showed large gaps, accompanied by a large number of vacuolar cells.

To illustrate the impacts of DGL interventions, histopathological alterations were classified into three distinct levels of liver damage. Results of the histopathology score are shown in Table 6. DGL administration tended to decrease the lesion score of the liver (*p* = 0.078).

### 3.6. Effect of DGL on Antioxidant Capacity

**Figure 2:**
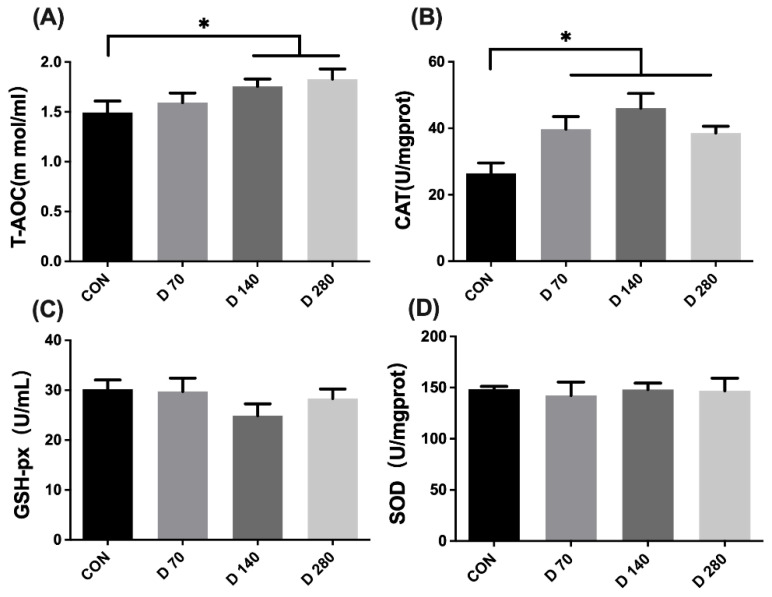
The influence of D-glucuronolactone on the antioxidant capacity of hens at the end of the experimental period. (**A**) T-AOC, total antioxidant capacity. (**B**) CAT, catalase. (**C**) GSH-Px, glutathione peroxidase. (**D**) SOD, superoxide dismutase. * Means between columns with an asterisk are different at *p* < 0.05.

Results of antioxidant capacity in the liver are shown in Figure 2. T-AOC and CAT content of the liver had significantly increased in hens that had DGL administration (*p* < 0.05). DGL administration showed no significant effect on the other parameters of antioxidant capacity in the liver (*p* > 0.05).

### 3.7. Effect of DGL on Inflammatory Response

**Figure 3:**
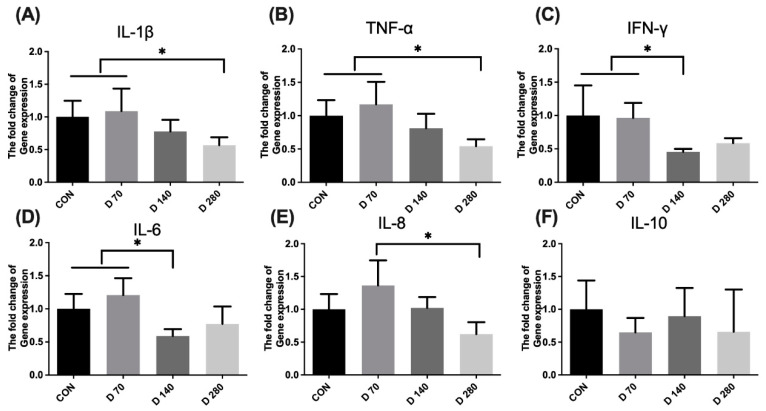
The influence of D-glucuronolactone on the inflammatory response of hens at the end of the experimental period. (**A**) IL-1β, interleukin 1beta. (**B**) TNF-α, tumor necrosis factor-alpha. (**C**) IFN-α, interferon-alpha. (**D**) IL-6, interleukin-6. (**E**) IL-8, interleukin-8. (**F**) IL-10, interleukin-10. * Means between columns with an asterisk are different at *p* < 0.05.

Results of antioxidant capacity in the liver are shown in Figure 3. DGL administration showed significant decreasing effects on the gene expression of pro-inflammatory factors in hens’ liver, such as TNF-α, IFN-γ, IL-1β, IL-6, and IL-8 (*p* < 0.05). There are no effects of DGL administration on IL-10 level in the liver (*p* > 0.05).

### 3.8. Effect of DGL on Cell Apoptosis

**Figure 4:**
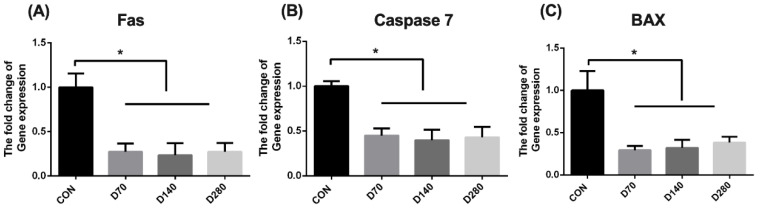
The influence of D-glucuronolactone on cell apoptosis-related genes expression of hens at the end of the experimental period. (**A**) Fas, factor-related apoptosis. (**B**) Caspase 7, Cysteine aspartic acid specific protease 7. (**C**) BAX, BCL 2 associated X Protein. * Means between columns with an asterisk are different at *p* < 0.05.

Results of cell apoptosis in the liver are shown in Figure 4. DGL administration significantly decreased the gene expression of Fas, Caspase 7, and BAX in the liver of hens (*p* < 0.05).

## 4. Discussion

The laying performance and egg quality show a gradual decline during the late peak period in hens, followed by a sharp decrease with advancing age [21]. Studies indicate that lipid peroxidation and protein oxidation are induced in the liver, hepatocyte growth and antioxidant recovery are inhibited, and liver tissue repair is impaired. These processes ultimately lead to reduced production performance [22,23]. To mitigate the decline and extend peak production, various additives have been used to alleviate liver stress in hens [19,24]. In this study, dietary DGL supplementation positively influenced the laying rate and egg mass after 12 weeks. Additionally, liver health improved with DGL supplementation, as evidenced by reduced lipid deposition and enhanced antioxidant capacity. Relief from inflammation and cell apoptosis was also observed.

DGL, known for its liver detoxification effects, has been used to protect against the poisoning from food, drugs, and endogenous metabolic toxicants produced in the liver [14]. Traditionally, DGL has been used as a hepatic antidote to alleviate symptoms of epidemic hepatitis and liver cirrhosis [25]. Serum ALT, AST, GGT, and TBIL levels, which serve as biomarkers of hepatic damage and hepatobiliary dysfunction in clinical evaluations, are sensitive indicators of liver health [26,27]. In this study, DGL administration significantly reduced serum AST and GGT activities, indicating its role in alleviating liver lesions in hens after the peak production period. Similar effects of DGL on humans have been reported [28]. Furthermore, histopathological results confirmed that DGL supplementation improved liver condition in hens. These findings align with recent studies showing that DGL protects liver health in rats exposed to drug-induced liver injury models [29,30] and in chicken hepatocytes [15]. These results suggest that DGL supplementation effectively supports liver health in hens with a high rate of egg-laying.

Moreover, DGL is widely added to energy drinks, which have a dominant presence in the drinks market. In these drinks, DGL is believed to prevent glycogen depletion by inhibiting other substances depleting muscle glycogen supplies [14,31]. These effects are hypothesized to attenuate fat deposition in the liver. In this study, dietary DGL supplementation demonstrated a significant reduction in liver lipid deposition, as reflected in parameters such as liver index, liver fat, TG content, and histopathological changes. This lipid-reducing effect was also observed in rats [32]. As a direct precursor of biosynthetic d-glucaric acid, oral administration of DGL increased d-glucaric acid excretion approximately 100-fold, while d-glucaric acid administration alone had minimal effect [33]. After oral administration, DGL is metabolized into nontoxic glucuronic acid, which conjugates with toxic metabolites from the liver and various drugs [34]. This process is facilitated by phase II conjugation enzymes, such as UDP-glucuronosyltransferase (UGT). The resulting conjugation metabolites are water-soluble and excreted through the gastrointestinal tract. In this process, lipids and fatty acids in the liver may conjugate with glucuronic acid and be excreted, reducing TG synthesis and deposition [35]. Meanwhile, glucuronic acid exhibits an inhibitory effect on α-amylase and α-glucosidase activities, which significantly improve glucose utilization, glycogen formation, and the activities of pyruvate kinase and hexokinase in insulin-resistant cells [13]. These mechanisms may explain the lipid-lowering effect of DGL in this trial.

Based on the lipid-lowering effect in the liver, our findings suggest that DGL may help prevent fatty liver syndrome (FLS) in hens under normal rearing conditions. As the most prevalent liver metabolic disorder, FLS negatively affects egg production and quality, potentially causing significant economic losses in the laying industry [23,36]. Lipid accumulation in the liver, along with injury, has been associated with hepatic and systemic inflammation. This inflammatory response may trigger liver blood vessel rupture and capsule damage, leading to the onset of FLS [37]. Meanwhile, excessive lipid accumulation in the liver can result in lipotoxicity and is strongly correlated with increased ROS and cell apoptosis [38]. Our results indicate that dietary DGL supplementation effectively reduces pro-inflammatory factor expression and inhibits cell apoptosis in the liver, along with enhanced antioxidant capacity. These findings align with recent studies showing that DGL protects hepatocytes from oxidative damage by scavenging free radicals both in vitro and in vivo [15,30]. The positive effects of DGL may involve the apoptosis pathway, as it appears to reduce apoptotic cell numbers following toxic exposure [39]. Oxidative stress induces apoptosis in ovarian granulosa cells, contributing to follicular atresia. Feed additives that improve antioxidant capacity may enhance laying performance [40,41]. Mitigating these effects potentially indicates the impact of DGL on laying performance.

In this study, one unanticipated finding was that DGL administration significantly deepened the brown color of eggshells and tended to increase eggshell strength. The eggshell color, a significant external attribute of eggs, directly indicates quality for consumers and correlates positively with shell quality [42]. Our results align with previous studies. Interestingly, the trial results revealed that adding any dose of DGL deepened eggshell color, which has not been reported previously. Among eggshell pigments, porphyrin IX likely plays a predominant role in the formation of the brown eggshell color [43,44]. Porphyrin IX is believed to originate from either free or aged erythrocytes through a degradation process in which the heme component of erythrocytes breaks down [45]. After degradation, the bilirubin glucuronidation reaction occurs in hens, providing raw materials for eggshell pigment formation [46]. We hypothesized that exogenous DGL addition improves this process, offering more materials for pigment deposition on eggshells. However, the underlying mechanisms remain unclear and require further investigation.

The results presented suggest that including the DGL may mitigate age-related liver damage in hens by decreasing liver fat deposition, regulating antioxidant capacity, and reducing inflammatory responses. These effects ultimately reduce the liver burden, increasing the laying rates and extending the peak laying period in hens. DGL appeared effective at dosages of 140–280 mg/kg, with the maximum effect observed at 280 mg/kg in this trial. The positive effects of DGL supplementation increased linearly with dosage. However, the potential benefits of higher dosages or longer DGL administration should be further explored for laying hens.

## 5. Conclusions

In conclusion, dietary DGL administration improved the laying performance and eggshell quality of hens during the late peak period. The mechanisms underlying these effects involve improvements in the liver health, including reductions in fat deposition, enhanced antioxidative capacity, modulation of inflammatory responses, and prevention of cell apoptosis. DGL may enhance the brown pigmentation of eggshells by increasing the availability of precursors for protoporphyrin synthesis. Additionally, the highest dose (280 mg/kg) of DGL elicited the best effects in this study for maintaining laying performance and promoting liver health in hens by influencing lipid metabolism. However, to precisely determine the optimal dosage, future research should explore whether administering higher doses could yield even greater benefits.

## Figures and Tables

**Table 1:** Ingredient and nutrient composition of the basal diet (air-dried basis, %).

Ingredients	Content (%)	Nutrient Levels ^3^	Content (%)
Corn	60.00	Metabolizable energy (MJ/kg)	11.34
43% Soybean meal	28.43	Crude protein	17.05
Soybean oil	1.00	Calcium	3.52
Limestone	8.30	Available phosphorus	0.44
Dicalcium phosphate	1.50	Lysine	0.83
DL-methionine(99%)	0.15	Cystine	0.24
NaCL	0.30	Methionine	0.41
70% Choline chloride	0.09		
Mineral premix ^1^	0.20		
Vitamin premix ^2^	0.03		
Total	100.00		

^1^ Mineral premix provided the following per kilogram of the diet: Cu, 20 mg; Fe, 144 mg; Zn, 144 mg; Mn, 120 mg; Se, 0.70 mg; I, 0.96 mg; Co, 1 mg. ^2^ Vitamin premix provided the following per kilogram of the diet: vitamin A, 13,500 IU; vitamin D3, 3900 IU; vitamin E, 30 IU; vitamin K3, 4.8 mg; vitamin B1, 3 mg; vitamin B2, 7.5 mg; vitamin B6, 6 mg; vitamin B12, 0.024 mg; folic acid, 1.5 mg; biotin, 0.18 mg; niacin, 45 mg; calcium pantothenate, 18 mg. ^3^ Nutrient levels were all measured except the metabolizable energy.

**Table 2:** Primer sequences for RT-PCR.

Items	Genes	Primer Sequences (5′-3′)	Gene Bank No.
Inflammatory response	IL-1β	F: CCGAGGAGCAGGGACTTT R: AGGACTGTGAGCGGGTGT	NM_204524.1
	TNF-α	F: CAGGACAGCCTATGCCAACAAG R: GGTTACAGGAAGGGCAACTCATC	NM_204267.1
	IFN-γ	F: CAAGCTCCCGATGAACGACTT R: AGTTGAGCACAGGAGGTCAT	NM_205149.1
	IL-6	F: TTTATGGAGAAGACCGTGAGG R: TGTGGCAGATTGGTAACAGAG	NM_204628.1
	IL-8	F: ATGAACGGCAAGCTTGGAGCTG R: TCCAAGCACACCTCTCTTCCATCC	NM_205498.1
	IL-10	F: GCTGAGGGTGAAGTTTGAG R: CAGGTGAAGAAGCGGTGA	NM_001004414.2
Cell apoptosis	Fas	F: TTGGACGAGTGTATGAGATG R:ACAGTGTCTGAAGTTGAAGT	XM_015288392.4
	Caspase 7	F: TCTCTGTGCTCTGTGCTA R:AAGGAATCTGCTTCTTCTCA	XM_046920622.1
	BAX	F:GTGATGGAGGTGACTGAAG R:AATCTGGTCCTGGCTGTT	XM_040693909.2
Reference gene	β-actin	F: TCAGGGTGTGATGGTTGGTATG R: TGTTCAATGGGGTACTTCAGGG	NM_205518.1

Abbreviations: IL-1β, interleukin 1beta; TNF-α, tumor necrosis factor-alpha; IFN-γ, interferon-gama; IL-6, interleukin-6; IL-8, interleukin-8; IL-10, interleukin-10; Fas, factor-related apoptosis; Caspase 7, Cysteine aspartic acid specific protease 7; BAX, BCL2 associated X Protein; β-actin, beta-actin.

**Table 3:** Effects of D-glucuronolactone on the laying performance of hens ^1^.

Items	Groups				SEM	*p*-Value		
	CON	D 70	D 140	D 280		ANOVA	Linear	Quadratic
Laying rate, %								
1–4 week	94.20	94.46	95.01	95.16	0.312	0.688	0.253	0.862
5–8 week	91.88	92.57	93.83	93.51	0.483	0.483	0.803	0.522
9–12 week	89.15 ^b^	91.92 ^ab^	91.98 ^ab^	95.16 ^a^	0.971	0.042	0.021	0.506
1–12 week	91.74 ^b^	92.98 ^ab^	93.71 ^ab^	94.61 ^a^	0.432	0.022	0.002	0.689
ADFI, g/hen/day								
1–4 week	119.95	119.29	122.50	120.33	0.338	0.101	0.712	0.388
5–8 week	114.93	114.25	116.40	114.75	0.452	0.686	0.445	0.627
9–12 week	124.10	120.01	122.62	125.80	0.701	0.538	0.508	0.606
1–12 week	119.75	117.50	120.53	118.96	0.312	0.199	0.891	0.762
AEW, g								
1–4 week	59.23	57.93	59.41	59.09	0.174	0.056	0.321	0.038
5–8 week	59.85 ^a^	58.22 ^b^	59.93 ^ab^	59.01 ^ab^	0.173	0.016	0.112	0.029
9–12 week	59.74 ^a^	57.77 ^b^	59.51 ^ab^	59.02 ^ab^	0.304	0.010	0.443	0.076
1–12 week	59.60 ^a^	57.97 ^b^	59.55 ^a^	59.03 ^ab^	0.155	0.008	0.199	0.015
Egg mass, g/day/hen								
1–4 week	55.79 ^ab^	54.72 ^b^	56.45 ^a^	56.23 ^a^	0.230	0.031	0.218	0.090
5–8 week	54.99 ^ab^	53.89 ^b^	56.23 ^b^	55.17 ^ab^	0.307	0.044	0.349	0.452
9–12 week	53.26	53.10	54.74	56.16	1.191	0.156	0.054	0.238
1–12 week	54.68 ^ab^	53.90 ^b^	55.80 ^a^	55.85 ^a^	0.282	0.022	0.039	0.109
FCR, feed(g)/egg(g)								
1–4 week	2.15	2.18	2.17	2.14	0.010	0.273	0.943	0.053
5–8 week	2.09	2.12	2.07	2.08	0.012	0.454	0.481	0.396
9–12 week	2.33	2.26	2.24	2.24	0.022	0.555	0.180	0.646
1–12 week	2.19	2.18	2.16	2.13	0.011	0.210	0.056	0.384
Mortality rate, %	0	0	0	0	——	——	——	——

^a,b^ Means with different superscripts within a column are different at *p* < 0.05. Abbreviations: CON, basal diet group; D 70, D 140, and D 280, basal diet supplemented with 70 mg/kg, 140 mg/kg, and 280 mg/kg of D-glucuronolactone, respectively. SEM = standard error of means. The same as tables below. ^1^ Results are means with *n* = 8 per treatment, the same as tables below.

**Table 4:** Effects of D-glucuronolactone on the egg quality parameters of hens.

Items	Groups				SEM	*p*-Value		
	CON	D 70	D 140	D 280		ANOVA	Linear	Quadratic
Egg shape Index ^1^	1.33	1.33	1.32	1.32	0.004	0.545	0.285	0.647
Shell strength, kg/cm^2^	3.84	4.33	4.37	4.60	0.097	0.082	0.091	0.452
Shell ratio ^2^, %	9.92	10.03	10.21	9.98	0.082	0.764	0.624	0.524
Shell thickness, μm	335.03	332.93	338.99	338.61	2.791	0.858	0.577	0.759
Shell color	27.79 ^a^	25.37 ^b^	25.40 ^b^	24.15 ^b^	0.392	0.004	<0.001	0.669
Yolk ratio ^3^, %	28.75	28.08	28.89	36.58	1.924	0.365	0.228	0.220
Yolk color	6.48	6.55	6.35	6.05	0.078	0.256	0.128	0.190
Albumen Ratio ^4^,%	61.33	61.89	60.91	53.44	1.920	0.371	0.218	0.232
Haugh unit	76.75	79.48	79.80	79.18	1.101	0.770	0.389	0.549

^a,b^ Means with different superscripts within a column are different at *p* < 0.05. ^1^ Egg shape index: egg transverse span/egg longitudinal span; ^2^ shell ratio: shell weight/egg weight; ^3^ yolk ratio: yolk weight/egg weight; ^4^ albumen ratio: albumen weight/egg weight.

**Table 5:** Effects of D-glucuronolactone on the blood biochemical parameters of hens.

Items	Groups				SEM	*p*-Value		
	CON	D 70	D 140	D 280		ANOVA	Linear	Quadratic
TBIL, U/L	0.45	0.39	0.46	0.56	0.104	0.953	0.733	0.650
ALT, U/L	2.88	3.25	4.50	3.38	0.445	0.623	0.462	0.598
AST, U/L	182.88 ^a^	150.75 ^ab^	142.63 ^b^	134.50 ^b^	5.868	0.013	0.001	0.536
ALP, U/L	29.00	28.50	41.71	25.29	2.030	0.424	0.534	0.862
AST/ALT	76.95	75.40	58.04	66.72	2.353	0.894	0.586	0.946
GGT, U/L	56.88 ^a^	44.25 ^ab^	42.75 ^b^	32.88 ^b^	3.503	0.029	<0.001	0.218

^a,b^ Means with different superscripts within a column are different at *p* < 0.05. Abbreviations: TBIL, total bilirubin; ALT, alanine aminotransferase; AST, aspartate aminotransferase; ALP, alkaline phosphatase; GGT, γ-glutamyl transpeptidase.

**Table 6:** Effects of D-glucuronolactone on the liver indexes, lipids, and histopathology scores of hens.

Items	Groups				SEM	*p*-Value		
	CON	D 70	D 140	D 280		ANOVA	Linear	Quadratic
Liver index,%	2.32 ^a^	2.19 ^ab^	1.94 ^b^	2.02 ^b^	0.051	0.030	0.198	0.007
Liver fat, %	20.13 ^a^	18.67 ^ab^	17.29 ^ab^	17.07 ^b^	0.429	0.018	0.001	0.179
TG, mmol/g prot	0.31 ^a^	0.30 ^a^	0.29 ^ab^	0.26 ^b^	0.009	0.036	0.231	0.425
TC, mmol/g prot	0.078	0.080	0.083	0.077	0.001	0.363	0.209	0.281
Histopathology score	1.50	1.63	2.01	2.62	0.151	0.078	0.018	0.264

^a,b^ Means with different superscripts within a column are different at *p* < 0.05.

## Data Availability

The authors will supply the relevant data in response to reasonable requests.

## Abbreviations

ADFI	average daily feed intake
AEW	average egg weight
ANOVA	a one-way analysis of variance
ALP	alkaline phosphatase
ALT	alanine aminotransferase
AST	aspartate aminotransferase
BAX	BCL2 Associated X Protein
Caspase 7	Cysteine aspartic acid specific protease 7
CAT	catalase
DGL	D-glucuronolactone
Fas	factor-related apoptosis
FCR	feed conversion ratio
GGT	glutamyl transferase
GSH-Px	glutathione peroxidase
HE	hematoxylin–eosin
IFN-γ	interferon-gamma
IL-10	interleukin-10
IL-1β	interleukin 1beta
IL-6	interleukin-6
IL-8	interleukin-8
ROS	reactive oxygen species
SEM	standard error of mean
SOD	superoxide dismutase
T-AOC	total antioxidant capacity
TBIL	total bilirubin
TC	total cholesterol
TG	triglyceride
TNF-α	tumor necrosis factor-alpha
β-actin	beta-actin
